# 

**Published:** 1830-07

**Authors:** 


### LONDON MEDICAL AND PHYSICAL JOURNAL.

EDITED BY

JOHN NORTH, ESQ. F.L.S.

MEMBER OF THE ROYAL COLLEGE OF SURGEONS,

AND OF THE MEDICAL AND CHIRURGICAL SOCIETY OF LONDON.

Dec no wanting Oct 2/77

S.G.O. INDEXED

(VOL. LXV.)

NEW SERIES, VOL. IX.

Et quoniam variant morbi, variabimus artes;
Mille mali species, mille saluti erunt.

Surgeon Genl's Office LIBRARY 45597

LONDON:

JOHN SOUTER, 73, ST. PAUL'S CHURCH-YARD;

AND TO BE HAD OF ALL THE MEDICAL BOOKSELLERS.

1831.

W1
LO 31

J. AND C. ADLARD, PRINTERS,
BARTHOLOMEW CLOSE.

### MONTHLY LIST OF MEDICAL BOOKS.

[*Medical Works cannot be entered on this List except a copy be sent for the purpose; the titles of Books having frequently been transmitted to us, as published, which have not appeared for weeks, or even months, after.*]

Additions in the Second Edition of the Influence of Climate, &c. By James Clark, M.D.—pp. 39.

We have already briefly mentioned the many improvements in the second edition of Dr. Clark's very interesting work: in our next Number we shall give an analysis of these additions.

Two Lectures on some of the Physical Signs of Diseases of the Chest; delivered before the Members of the Portsmouth Philosophical Society in January 1829. By John Forbes, M.D. F.R.S. &c.—1830.

A Manual of Descriptive Anatomy, by Cloquet. Translated by Thomas King, Surgeon.—Dumas, Leicester square. Nos. II. III. and IV.

This excellent work cannot fail to be supported by medical students. Mr. King has conferred a great benefit upon his junior brethren, by affording them so good an opportunity of acquiring a perfect knowledge of anatomy and of the French language at the same time.

An Inquiry concerning the Indications of Insanity; with Suggestions for the better Protection and Care of the Insane. By John Conolly, M.D. Professor of Medicine in the University of London.—Taylor, 1830. 8vo. pp. 496.

Auli Cornelii Celsi de Re Medica. Libri octo. Editio nova. Ex recensione Lev. Targæ, curante C. F. Collier, M.D. Accedit Lexicon Celsianum breve. Vols. III. and IV.—32mo. Highley, London. With an English Translation.

Dr. Collier will not think we slight the useful task he has performed, if we limit ourselves to a general expression of our approbation of the work. It cannot fail to be of much assistance to medical students.

Specimen of an Introduction to the Study of Anatomy. By James Paxton, Surgeon, and author of Illustrations of Paley's Natural Theology.

Mr. Paxton's work will very shortly be published, and, judging from the specimen we have received, we cannot doubt that it will be the favorite book of study with young anatomists. The plates are very well executed; the references clear and distinct; the descriptive text concise and correct. The plan Mr. Paxton has adopted of giving the engraving and its description on the same page will materially enhance the value of the work to the student.

On Canine Madness; comprising the Symptoms, Post-mortem Appearances, Nature, Origin, and Preventive and Curative Treatment of Rabies in the Dog, and other Domestic Animals. By W. Gouatt, V.S. and F.Z.S. &c.—8vo. pp. 52. Longman, London, 1830.

Remarks on the Disease called Hydrophobia; Prophylactic and Curative. By John Murray, F.S.A. F.L.S. F.H.S. F.G.S. &c.—8vo. pp. 86. Longman, London, 1830.

